# Editorial: Skin - confluence of vertebrate host defences, arthropod vectors, and vector-borne pathogens

**DOI:** 10.3389/fimmu.2025.1688830

**Published:** 2025-09-11

**Authors:** Sukanya Narasimhan, Maria Kazimirova, Stephen Wikel

**Affiliations:** ^1^ Section of Infectious Diseases, Department of Internal Medicine, Yale School of Medicine, New Haven, CT, United States; ^2^ Institute of Zoology, Slovak Academy of Sciences, Bratislava, Slovakia; ^3^ Department of Medical Sciences, Frank H. Netter, M.D. School of Medicine, Quinnipiac University, Hamden, CT, United States

**Keywords:** vector-pathogen interactions, arthropod vector-skin interactions, arthropod saliva, host skin immunity, arthropod vector-host skin interface

Arthropod blood feeding, pathogen transmission, nuisance, and biting related injury are long-standing and increasingly important global threats to human and animal health. Arthropod interactions with vertebrate hosts occur at the cutaneous interface, a structural and immunological barrier rich in interconnected somatosensory elements, multiple resident and migrating immune cell types, and a complex, changing array of soluble response mediators (1–3). Vertebrate skin presents a mixed milieu of attraction and defense elements for the arthropod vector. The skin with its microbiome, metabolites, and odorants provides the chemical cues critical for determining vector-host preference (4–6). The skin microbiome may also set the immunological stage that determines critical host defense responses that the vector encounters during its treacherous mission of taking a bloodmeal and that the vector-borne pathogen encounters during its transmission and establishment in the host (7–9).

Blood feeding arthropods possess mouthpart structures and feeding strategies that are divided into the two broad categories of obtaining a blood meal from within the lumen of a blood vessel, solenophages, or creating a pool of blood by lacerating dermal blood vessels, telmophages (10, 11). Blood meal uptake occurs over the course of minutes for mosquitoes, biting flies, fleas, bed bugs, and triatomines; one to two hours for argasid ticks, and days to more than a week for ixodid ticks that also secrete attachment cement to aid in securing themselves to the bite site (12–14). Regardless of the mechanism or duration of feeding, successful acquisition of a blood meal is dependent not just upon the diverse immunomodulatory components of arthropod saliva secreted into the skin at the bite site during feeding, but also critically influenced by host defense responses to the vector saliva molecules deposited in the skin. The tug-of-war interactions that ensue between the vector and host skin defenses shape the evolution of vector-host relationships (15) resulting in host resistance or susceptibility to the vector.

Exploring the dynamics of vector-host interactions Kamran et al. describe efforts to define host biomarkers of ectoparasite resistance. The study focuses on the important veterinary pest *Haematobia irritans exigua*, buffalo fly, close relative of *Haematobia irritans irritans*, horn fly, both of whom are blood feeding dipterans responsible for economic losses estimated for horn fly in the United States to be one billion dollars annually (16). Buffalo flies are obligate blood feeders taking 20 to 40 blood meals per day (17). Bites of these flies cause severe cutaneous irritation resulting in open sores. To define biomarkers associated with enhanced fly resistance, the authors used LC-MS/MS to compare the serum proteome profile of Brangus cattle that supported high (HF) or low Buffalo fly (LF) infestations and demonstrated that proteins associated with wound healing, phagocytosis and coagulation were significantly increased in LF cattle compared to that in HF cattle. These observations offer additional biomarkers that synergize with ongoing efforts to breed cattle with increased disease resistance (18, 19).

Paine et al. examine tick transmission of Powassan virus, a neuroinvasive virus of public health importance, to vertebrate hosts under controlled, laboratory conditions. Studying this virus by tick transmission is essential due to the role tick saliva molecules play in facilitating host neuroinvasion by Powassan virus (20). Furthermore, Powassan virus is unique among *Ixodes scapularis* transmitted infectious agents in that transmission occurs as rapidly as fifteen minutes after tick attachment to the host (21), while other *I. scapularis*-transmitted pathogens require hours to days of blood feeding before successful transmission occurs (28). Paine et al. provide insights into how infestation with *Ixodes scapularis* impacts cutaneous global transcriptomic changes at the tick bite site during the earliest timepoints for Powassan virus transmission.

Advances in proteomics and genomics are facilitating a molecular understanding of how vector saliva molecules interact with host resident and migrating cells, signaling molecules, and receptors during and following blood feeding to provide novel insights into the interactions between vertebrate skin, arthropod vectors, and vector-borne pathogens (22, 23). Since the tick remains attached to the mammalian host for hours to days, depending on the tick species, the tick needs to modulate cutaneous innate and adaptive immune responses at the tick-host interface. Kleissl et al. provide an insightful review on tick saliva that examines how the tick exploits an array of biological functions of salivary proteins, peptides, small molecules, and non-coding RNAs in tick saliva to perform an astonishing variety of “tricks” at the host skin-vector interface that are critical to successful blood feeding.

Highlighting the importance of pharmacological activities in tick saliva, Molari et al. examine protease inhibitors, a predominant super family of proteins secreted in tick saliva (24). The study focuses on the cystatin family of protease inhibitors that inhibit cysteine proteases involved in multiple host defense response pathways including neutrophil recruitment, antigen processing and presentation, and apoptosis (25). The authors demonstrate that Amblyostatin-1, a secreted salivary cystatin from *Amblyomma sculptum*, a neotropical hard tick that is a vector of *Rickettsia rickettsii*, the agent of Rocky Mountain/Brazilian spotted fever, inhibits Cathepsin-S, a cysteine protease crucial for proper antigen loading and presentation on MHC-II by dendritic cells (26). Amblyostatin-1 impairs the function of dendritic cells (DCs) in the mammalian skin and in-turn the development of antibody responses to salivary antigens. Interestingly, Amblyostatin-1 also promotes the secretion of IL-10, an anti-inflammatory cytokine, by DCs. Exploiting a mouse model of carrageenan-induced inflammation (27), the authors show that Amblyostatin-1 reduced edema and neutrophil recruitment-induced cutaneous inflammation. Amblyostatin-like molecules represent exciting opportunities for the development of novel therapeutics for inflammatory diseases in humans.

The molecular repertoire of the saliva of hematophagous arthropod vectors evolved over millions of years to perfect the pharmacological functions that ensure successful blood feeding. Undoubtedly, evolution continues within, among, and across all parties in the hematophagous arthropod –host –arthropod transmitted pathogens triad. While our understanding of these relationships has advanced significantly during the past 50 years, we are only beginning to realize the multifaceted complexity, roles, dynamic crosstalk, and beauty of these interactions.
